# MRI-quantified left atrial epicardial adipose tissue predicts atrial fibrillation recurrence following catheter ablation

**DOI:** 10.3389/fcvm.2022.1045742

**Published:** 2022-12-02

**Authors:** Yaacoub Chahine, Fima Macheret, Karen Ordovas, Joonseok Kim, Patrick M. Boyle, Nazem Akoum

**Affiliations:** ^1^Division of Cardiology, University of Washington, Seattle, WA, United States; ^2^Department of Radiology, University of Washington, Seattle, WA, United States; ^3^Department of Bioengineering, University of Washington, Seattle, WA, United States; ^4^Institute for Stem Cell and Regenerative Medicine, University of Washington, Seattle, WA, United States; ^5^Center for Cardiovascular Biology, University of Washington, Seattle, WA, United States

**Keywords:** epicardial adipose tissue, catheter ablation, cardiac magnetic resonance, atrial fibrillation, Dixon sequence

## Abstract

**Background:**

Epicardial adipose tissue (EAT) plays a significant role in promoting atrial fibrillation (AF) due to its proinflammatory properties and anatomic proximity to the myocardium. We sought to assess whether left atrial (LA) EAT volume is associated with AF recurrence following catheter ablation.

**Methods:**

EAT was assessed *via* the 3D MRI Dixon sequence in 101 patients undergoing AF ablation. Patients were followed for arrhythmia recurrence.

**Results:**

During an average follow-up period of 1 year, post-ablation AF recurrence occurred in 31 (30.7%) patients. LA EAT index was higher in those with compared to without recurrence (20.7 [16.9, 30.4] vs. 13.7 [10.5, 20.1] mL/m^2^, *p* < 0.001), and so was LA volume index (66 [52.6, 77.5] vs. 49.9 [37.7, 61.8] mL/m^2^, *p* = 0.001). Cox regression analysis showed LA EAT (HR = 1.089; 95% CI: [1.049–1.131], *p* < 0.001) to be an independent predictor of post-ablation AF recurrence. The ROC curve for LA EAT index in the prediction of AF recurrence had an AUC of 0.77 (95% CI 0.68–0.86, *p* < 0.001) and showed an optimal cutoff value of 14.29 mL/m^2^ to identify patients at risk of post-ablation AF recurrence. Integrating LA EAT with clinical risk factors improved prediction of AF recurrence (AUC increased from 0.65 to 0.79, DeLong test *p* = 0.044). Kaplan-Meier analysis for recurrence-free survival showed a significant difference between two groups of patients identified by the optimal LA EAT index cutoff of 14.29 mL/m^2^ (log rank = 14.79; *p* < 0.001).

**Conclusion:**

EAT quantified using cardiac MRI, a reproducible and widely accessible imaging parameter, is a strong and independent predictor of post-ablation AF recurrence.

## Introduction

Atrial fibrillation (AF) remains the most prevalent clinical arrhythmia in the world with an associated significant morbidity and mortality (1). The prevalence of AF is projected to increase in the coming decades (2). Studies have demonstrated that obesity is closely associated with AF incidence (3). Epicardial adipose tissue (EAT) is thought to be a crucial factor mediating cardiovascular disease in obesity, it has been linked to increased risk of atherosclerotic disease (4), ventricular dysfunction (5), coronary artery disease (6) and atrial fibrillation (7). EAT is metabolically active visceral adipose tissue, located between the myocardium and the pericardium; it shares a micro-circulatory environment with the myocardium (originating from the coronary circulation) and it is in direct contact with the myocardium without any fascial separation. Despite being phenotypically similar to white adipose tissue, EAT highly expresses Uncoupling Protein 1, suggesting a similar function to brown adipose tissue. These biological and functional characteristics have warranted the application of a new nomenclature to EAT which is beige adipose tissue (8).

The relationship between EAT and AF is likely multifactorial. EAT can promote a localized inflammatory reaction within the myocardium through proinflammatory adipokines (9). It is also thought to secrete profibrotic factors like tumor growth factor β, matrix metalloproteinases, and activin A, all of which can induce fibrotic remodeling within the myocardial wall with a resultant electrical heterogeneity and local conduction block forming a substrate for AF (10, 11). LA EAT was shown to correlate with fibrotic remodeling in the atrial wall (12).

Catheter ablation is widely performed for rhythm control of symptomatic paroxysmal or persistent AF with variable results in complete suppression of arrhythmia recurrence, especially in obese patients (13). The relationship between the volume of EAT and AF recurrence after ablation is under explored. Previous studies used computed tomography or echocardiography to assess LA EAT burden and its relation to AF recurrence post-ablation (14) but to date no data is yet available regarding the MRI quantification of LA EAT and recurrence of AF post-ablation. We aimed to assess the predictive role of cardiac MRI-quantified LA EAT volume, using the 3D Dixon sequence, in AF recurrence following catheter ablation.

## Methods

### Study design and population

This is an observational study that included patients with paroxysmal or persistent AF undergoing their first catheter ablation for AF at the University of Washington Medical Center (UWMC), who also underwent a cardiac MRI for EAT quantification. Patients were consecutively included between January 2020 and September 2021.This study was approved by the Institutional Review Board (IRB) of the University of Washington and all participants provided verbal consent (HSD#6058). The target of the catheter ablation was pulmonary vein isolation and no additional ablation lesions outside the pulmonary vein antral region were delivered. Exclusion criteria included prior catheter ablation, contraindications to MRI or gadolinium-based contrast including implantable electric devices, severe claustrophobia, and renal dysfunction. Comorbidities and medications at the time of the initial visit were determined using electronic medical record review. Study data were collected and managed using the REDCap system hosted at the University of Washington (15, 16).

### MRI protocol

All images were obtained using a Philips Medical System Ingenia 1.5 T clinical scanner. To assess EAT, a 3D respiration-navigated, ECG-gated Dixon sequence was implemented into the cardiac imaging protocol at the UWMC with the following parameters: 1.5 mm slice thickness, repetition time (TR) = 5.4 ms, echo time 1/echo time 2 = 1.8/4.0 ms, flip angle (α) = 15°, voxel size = 1.5 × 1.5 × 3.0 mm^3^ (reconstructed to 1.0 × 1.0 × 1.5 mm^3^), parallel imaging factor (SENSE) = 1.5 in both phase encoding directions and water fat shift = 0.16 pixel. Arrhythmia rejection was applied, the T2 preparation duration was 50 ms and the acquisition window was 100 to 156 ms. For fibrosis evaluation, LGE-MRI was acquired following the methods previously described (17). Briefly, scans were performed 20 min after contrast injection, using a 3D inversion-recovery, respiration-navigated, ECG-gated, gradient echo pulse sequence.

### Image analysis

**Figure 1:**
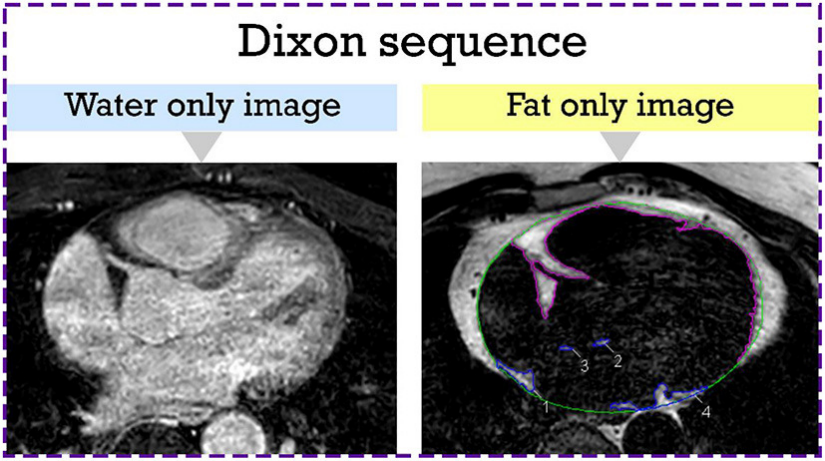
Epicardial adipose tissue segmentation from the axial Dixon MRI sequence. Hyperintense regions around the LA which represent LA EAT are manually contoured in blue. The rest of the EAT is contoured in purple. The pericardium is outlined in green. EAT, epicardial adipose tissue; LA, left atrial.

Dixon images were exported using the CVI42 software (Circle Cardiovascular Imaging Inc., version 5.6, Calgary, AB). EAT was defined as the adipose tissue located between the visceral layer of the pericardium and outer surface of the myocardium. CVI42 contouring tools were used to segment areas of fat in the axial view (Figure 1). Areas of fat were manually traced on consecutive transaxial images and multiplied by the slice thickness to calculate EAT volume. LA EAT was characterized by hyperintense areas around the LA starting cranially from the bifurcation of the pulmonary artery to the mitral annulus caudally. The pericardium was outlined in the axial images and used as the external border for EAT. Total EAT was calculated as the sum of atrial and ventricular EAT in all slices from the pulmonary artery bifurcation to the ventricular apex. Pre-ablation LGE-MRI based fibrosis quantification was carried out by a third-party image processing service (Merisight, Marrek Inc., Salt Lake City, UT) using previously described methods (17).

### Catheter ablation

AF ablation indications were in accordance with current guidelines (13). All ablation procedures were performed under general anesthesia. Transseptal puncture was performed under fluoroscopic and intracardiac echocardiographic guidance. Patients were anticoagulated for 3 weeks prior to the procedure. Activated clotting time was maintained between 350 and 450 s throughout the procedure by unfractionated heparin. Pulmonary vein isolation (PVI) was the goal of the ablation procedure. The choice of radiofrequency or cryoballoon ablation was left to the discretion of the operator for these first-time patients. Radiofrequency ablation was performed using a wide area circumferential ablation approach targeting pulmonary vein isolation. No additional ablation lesions outside the pulmonary vein antral region were delivered. Cryoballoon ablation also targeted pulmonary vein isolation through sequential ablation delivered using a Medtronic Artic Front second generation 28 mm ablation catheter.

### Follow-up and arrhythmia recurrence

This is a retrospective study based on the regular follow-up of AF patients undergoing catheter ablation at the UWMC. Patients were followed at the UWMC clinic and had 7-day Holter monitoring at 3 months in addition to ambulatory ECG monitoring at 6, and 12 months following the ablation procedure. Arrhythmia recurrence was defined by at least 30 s of documented atrial arrhythmia after observing a 90-day blanking period (18). Patients with self-reported symptoms were evaluated with additional ECGs and ambulatory monitors.

### Statistical analysis

Categorical variables are expressed as percentages. Continuous variables are reported as mean ± SD if normally distributed, or median and interquartile range if not. Normal distribution was assessed by the Shapiro-Wilk test. For continuous variables, differences between two groups were compared using Student's *t-*test (for parametric variables) or the Mann-Whitney U test (for non-parametric variables). Categorical variables were compared using the chi-square test or Fischer's exact test. Inter and intra-observer reliability analysis was performed on a subset of 10 patients.

Univariable and multivariable Cox proportional hazards regression models were used to test the effect of the explanatory variables on the post-ablation AF recurrence. Variables with *p* < 0.1 in the univariable analysis were entered as covariates in the multivariable analysis. Two multivariable analysis models were created, one with LA EAT index as a continuous variable (model 1) and one with LA EAT index as a categorical variable using the cutoff value of 14.29 mL/m^2^ (model 2). Results were reported as hazard ratio with a 95% confidence interval (CI). A receiver operating characteristic (ROC) curve analysis and the Youden's J statistic (sensitivity + specificity – 1) were used to determine the best cutoff value of the LA EAT index for the prediction of AF recurrence post ablation. A multivariable logistic regression model with the clinical characteristics age, BMI, AF type and hypertension was created. In the clinical model, all variables were given the same weight. Age and BMI were included as continuous variables, whereas AF type and hypertension were included as categorical variables (paroxysmal or persistent AF, presence or absence of hypertension). Subsequently, LA EAT index was integrated to the clinical model to assess whether AF recurrence discrimination improved. The areas under the ROC curve were calculated and compared using the DeLong test for the two predictive testing strategies of AF recurrence. AF recurrence-free survival was then estimated by the Kaplan-Meier method and compared using a log-rank test between two study groups. All tests were 2-sided and a *p* < 0.05 was considered statistically significant. Statistical analysis was performed using SPSS statistical software (version 26.0, International Business Machines Inc) and R Statistical Software version 4.1.1 (R Foundation for Statistical Computing) (19).

## Results

A total of 101 patients were included in the study. The median age of our study population was 67 [57.5, 73] years (72.3% male). The average BMI was 28.07 [24.96, 34.32] kg/m^2^. 59.4% of the participants had paroxysmal AF and the rest had persistent AF (40.6%). The average LA volume index was 54.2 [41.23, 67.51] mL/m^2^ while the fibrosis burden was 16.9% [11.2, 24] of the LA wall. The median LA EAT volume was 34.65 [23.84, 47.73] mL while the median LA EAT index, adjusted to the body surface area (BSA), was 16.89 [12.24, 22.02] mL/m^2^.

The intraclass correlation coefficients for inter-observer and intra-observer reliability for EAT measurements were 0.927 (95% CI, 0.734–0.981) and 0.968 (95% CI, 0.877–0.992), respectively. The Bland-Altman analysis for inter-observer reliability revealed a good agreement between the results of EAT measurements performed by two different readers. The mean difference between the observers was −0.59 mL with 95% limits of agreement of (−25.11; 23.93) mL for EAT volume. The Bland-Altman analysis for intra-observer reliability revealed a good agreement between the results of repeated EAT measurements performed by the same reader. The mean difference between the repeated measurements was −0.29 mL with 95% limits of agreement of (−16.75; 16.17) mL for EAT volume.

Table 1 shows the baseline characteristics for patients with and without AF recurrence post-ablation. 75 (74.3%) patients underwent RF ablation, whereas 26 (25.7%) patients underwent cryoballoon ablation. No patients were lost to follow-up. There were 31 (30.7%) patients who developed AF recurrence after catheter ablation during a median follow-up duration of 365 days [307, 448]. The median time for AF recurrence following CA was 136 days [96, 267]. Compared to patients without arrhythmia recurrence, patients with recurrence had a significantly larger LA volume index (66 mL/m^2^ vs. 49.9 mL/m^2^, *p* = 0.001); and a higher LA EAT index (20.7 mL/m^2^ vs. 13.7 mL/m^2^, *p* < 0.001). We adjusted LA EAT volume for LA volume to rule out collinearity between the two, and the ratio was significantly higher in patients who had a recurrence of AF post ablation (0.34 [0.25, 0.51] vs. 0.29 [0.21, 0.37], *p* = 0.035).

**Table 1:** Baseline characteristics of patients with and without post ablation arrhythmia recurrence.

**Variable, *n* (%)**	**No recurrent arrhythmia (*n =* 70)**	**Recurrent arrhythmia (*n =* 31)**	***p*-value**
Age (years)	66 [57, 71.25]	68 [59, 74]	0.306
BMI (kg/m^2^)	27.7 [24.75, 34.3]	29.6 [25.9, 34.9]	0.327
BSA (m^2^)	2.1 ± 0.32	2.16 ± 0.28	0.39
Sex, male	53 (75.7%)	20 (64.5%)	0.246
Hypertension	34 (48.6%)	16 (51.6%)	0.778
Coronary artery disease	16 (22.9%)	7 (22.6%)	0.976
Congestive heart failure	18 (25.7%)	12 (38.7%)	0.187
Obstructive sleep apnea	16 (22.9%)	7 (22.6%)	0.976
Stroke	9 (12.9%)	1 (3.2%)	0.169
Hyperlipidemia	25 (35.7%)	14 (45.2%)	0.368
Diabetes mellitus	12 (17.1%)	8 (25.8%)	0.314
Non-paroxysmal AF	25 (35.7%)	16 (51.6%)	0.133
RF ablation	50 (71.4%)	25 (80.6%)	0.329
LA EAT index (mL/m^2^)	13.7 [10.5, 20.1]	20.7 [16.9, 30.4]	< 0.001
Total EAT index (mL/m^2^)	73.9 ± 28.3	85 ± 26.2	0.066
LA volume index (mL/m^2^)	49.9 [37.7, 61.8]	66 [52.6, 77.5]	0.001
LA fibrosis (%)	16.1 [9.7, 21.6]	18.6 [13.25, 26.65]	0.076
LA EAT volume/LA volume ratio	0.29 [0.21, 0.37]	0.34 [0.25, 0.51]	0.035
AF duration prior to ablation (months)	20 [5, 45.75]	35 [9, 55]	0.118

BMI, body mass index; BSA, body surface area; AF, atrial fibrillation; RF, radiofrequency; LA, left atrial; EAT, epicardial adipose tissue.

There was a trend toward a higher fibrosis burden in patients who had an AF recurrence without reaching statistical significance in this population (18.6 vs. 16.1%, *p* = 0.076). A similar proportion of patients received radiofrequency ablation in the recurrent vs. non-recurrent groups (80.6 vs. 71.4% respectively, *p* = 0.329). There was a trend toward a longer duration of AF prior to catheter ablation in the group of patients who had recurrent AF, but without reaching statistical significance (35 [9, 55] vs. 20 [5, 45.75] months, *p* = 0.118).

Univariable and multivariable time-dependent Cox regression models analyzing the predictors of AF recurrence are summarized in Table 2. Univariable Cox proportional hazard modeling showed that a larger LA volume (HR, 1.019; 95%CI, 1.005–1.034; *p* = 0.009) and a higher LA EAT index (HR, 1.089; 95% CI, 1.055–1.124; *p* < 0.001) were associated with increased AF recurrence in the follow-up period. There also was a trend toward persistent AF in patients who had a recurrence (HR, 1.917; 95% CI, 0.945–3.888; *p* = 0.071). Multivariable Cox regression model 1 showed that only increased LA EAT index (HR, 1.089; 95% CI, 1.049–1.131; *p* < 0.001) was an independent predictor of AF recurrence post ablation after adjusting for other variables.

**Table 2:** Univariable and multivariable predictors of arrhythmia free survival with a Cox regression.

	**Univariable analysis**		**Multivariable analysis 1**		**Multivariable analysis 2**	
	**HR (95% CI)**	***p*-value**	**HR (95% CI)**	***p*-value**	**HR (95% CI)**	***p*-value**
Age (years)	1.011 (0.977–1.047)	0.526				
BMI (kg/m^2^)	1.032 (0.982–1.085)	0.213				
BSA (m^2^)	1.666 (0.561–4.951)	0.358				
Sex, male	0.576 (0.274–1.211)	0.146				
Hypertension	1.005 (0.496–2.035)	0.989				
Coronary artery disease	0.831 (0.357–1.935)	0.668				
Congestive heart failure	1.571 (0.762–3.238)	0.221				
Obstructive sleep apnea	1.056 (0.455–2.453)	0.899				
Stroke	0.273 (0.037–2.007)	0.202				
Hyperlipidemia	1.235 (0.608–2.507)	0.559				
Diabetes mellitus	1.497 (0.669–3.35)	0.326				
Non-paroxysmal AF	1.917 (0.945–3.888)	0.071	0.781 (0.333 to 1.83)	0.569	1.174 (0.559, 2.464)	0.671
RF ablation	1.794 (0.733–4.39)	0.201				
LA EAT index (mL/m^2^)	1.089 (1.055–1.124)	< 0.001	1.089 (1.049 to 1.131)	< 0.001		
LA EAT index >14.29 mL/m^2^	7.331 (2.227–24.135)	0.001			6.397 (1.87, 21.88)	0.003
Total EAT index (mL/m^2^)	1.009 (0.996–1.022)	0.17				
LA volume index (mL/m^2^)	1.019 (1.005–1.034)	0.009	1.008 (0.991 to 1.025)	0.343	1.009 (0.993, 1.025)	0.281
LA fibrosis (%)	1.038 (0.991–1.087)	0.119				
AF duration prior to ablation (months)	1.007 (0.996, 1.017)	0.203				

BMI, body mass index; BSA, body surface area; AF, atrial fibrillation; RF, radiofrequency; LA, left atrial; EAT, epicardial adipose tissue.

**Figure 2:**
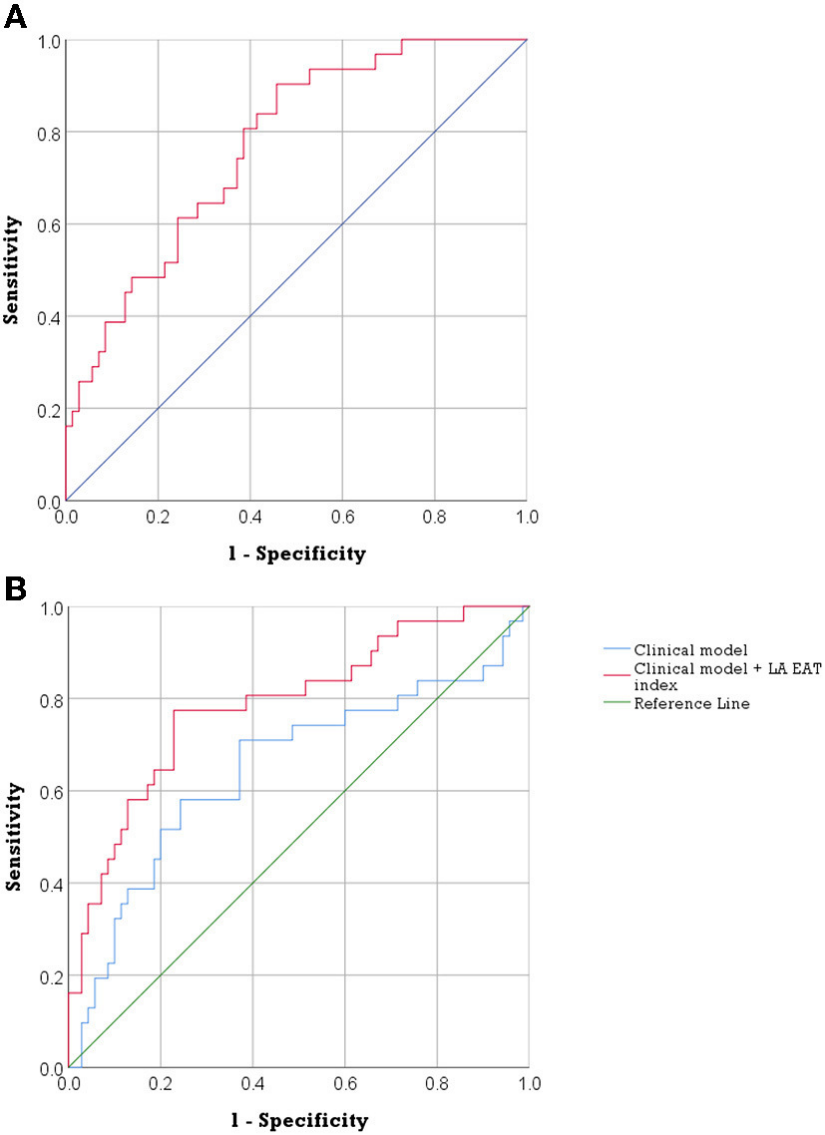
The predictive accuracy of LA EAT in post ablation AF recurrence. **(A)** ROC curve of LA EAT index in the prediction of AF recurrence. (AUC = 0.771, 95% confidence interval 0.68–0.86, *p* < 0.001). **(B)** ROC curves describing the discriminatory performance of integration of LA EAT index with the clinical model in the prediction of AF recurrence [AUC 0.788 (95% CI, 0.69–0.89) compared to 0.648 (95%CI, 0.52–0.78), DeLong test *p* = 0.044]. AF, atrial fibrillation; AUC, area under the curve; EAT, epicardial adipose tissue; LA, left atrial; ROC, receiver operating characteristic.

Figure 2 shows (A) the ROC curve for LA EAT index in the prediction of AF recurrence and (B) ROC curves comparing the effect of integrating LA EAT with the clinical model to the clinical model alone on predicting post-ablation AF recurrence. The ROC curve in Figure 2A showed an optimal cutoff value of 14.29 mL/m^2^ to identify patients at risk of AF recurrence post ablation (Youden's J statistic = 0.446). This cutoff had a sensitivity of 90.3%, specificity of 54.3%, positive predictive value of 46.7%, and negative predictive value of 92.7% in predicting AF recurrence. The area under the ROC curve was 0.771 (95% confidence interval 0.68–0.86, *p* < 0.001). Having an LA EAT index above the optimal cutoff of 14.29 mL/m^2^ had a univariate HR 7.33 (95% CI 2.23–24.14, *p* = 0.001) for prediction of AF recurrence. Multivariable Cox regression model 2 showed that LA EAT index >14.29 mL/m^2^ was an independent predictor of AF recurrence (HR, 6.397; 95% CI, 1.87–21.88; *p* = 0.003).

Integration of LA EAT index and the clinical model (including age, BMI, AF type and HTN) improved discriminatory performance to predict AF recurrence compared to the clinical model alone, area under the curve 0.788 (95% CI, 0.69–0.89) compared to 0.648 (95%CI, 0.52–0.78), DeLong test *p* = 0.044 (Figure 2B).

**Figure 3:**
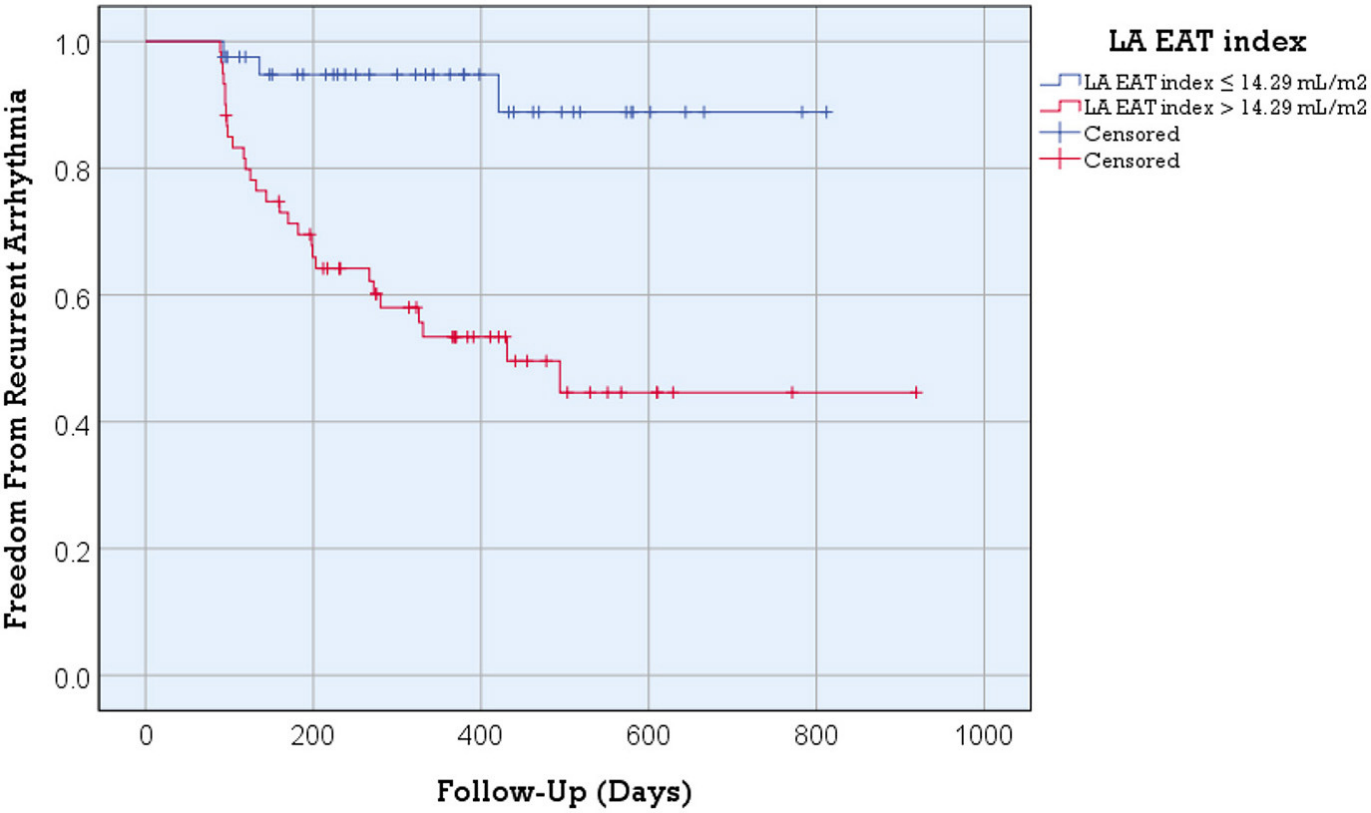
Kaplan-Meier arrhythmia free survival analysis for post ablation AF recurrence according to the LA EAT index. Better arrhythmia free survival was detected in patients with an LA EAT index ≤ 14.29 mL/m^2^ (log rank = 14.79; *p*-value < 0.001). Recurrent arrhythmia was observed in 7.3% of patients with an LA EAT index >14.29 mL/m^2^ compared to 46.7% of patients with an LA EAT index > 14.29 mL/m^2^ (*p*-value <0.001).

The Kaplan-Meier curve for freedom from arrhythmia recurrence according to the LA EAT index (Figure 3) showed a significant difference in the overall post ablation AF recurrence between the two groups of patients with the optimal LA EAT index cutoff value of ≤ 14.29 mL/m^2^ vs. >14.29 mL/m^2^ (log rank = 14.79; *p*-value < 0.001).

## Discussion

In this study, we show a clear and independent relationship between LA EAT and arrhythmia recurrence after catheter ablation for AF. A higher LA EAT index was predictive of AF recurrence independently of other AF risk factors, and the integration of LA EAT to clinical risk factors of AF improved discriminatory performance in predicting AF recurrence.

EAT can be quantified in a non-invasive manner using computed tomography (CT), cardiac magnetic resonance and echocardiography. The increasing availability and use of these non-invasive imaging modalities can lead to a simultaneous assessment of EAT in scans taken for multiple indications. Cardiac ultrasound is the most accessible and affordable imaging modality to assess the fat surrounding the heart, however measuring EAT thickness over the anterior right ventricle provides only an estimate of the overall cardiac EAT with no accurate volumetric assessment. This approach therefore lacks the sensitivity required to differentiate between types of cardiac fat. The high spatial resolution of cardiac CT allows greater accuracy in EAT quantification, and defining attenuation value ranges can allow for manual or semi-automatic segmentation of epicardial fat. Cardiac MR is currently considered the gold standard in imaging any type of adipose tissue because it has excellent spatial resolution but lacks the ionizing radiation hazards associated with CT. Recently, fat-water separation sequences (e.g., Dixon) proved valuable in detecting pericardial outlines and accurately quantifying EAT (12, 20).

In our study, EAT imaged using the Dixon MRI sequence was highly predictive of AF recurrence following ablation. This observation is consistent with results from previous studies using different imaging techniques to quantify EAT. Echocardiography derived EAT thickness over the right ventricle in parasternal long axis view was shown to be a useful parameter in predicting recurrences post ablation (21). Epicardial fat thickness measured on the axial view of the long side of the right ventricular free wall identified by cardiac MRI using T2 sequences was a predictive factor for AF recurrence after a first pulmonary vein isolation using second generation cryoballoon ablation (22). A recent systematic review and meta-analysis outlined the relationship between epicardial fat tissue imaged using echocardiography and CT with AF recurrence and found that total EAT volume, LA EAT volume, and epicardial fat thickness were associated with recurrence in patients undergoing AF ablation (14). Whereas a recent paper by Hammache et al. suggested that, unlike individuals with persistent AF, EAT parameters in patients undergoing paroxysmal AF ablation are not associated with AF recurrence (23).

Nalliah et al. showed that local EAT content was associated with decreased conduction velocity and more complex activation patterns, accompanied by increased myocardial fibrosis and sarcolemmal lateralization of connexin 40 gap junction proteins (24). Mahajan et al. showed that obesity was associated with increased EAT volume and atrial electroanatomical remodeling; in particular low voltage areas, slowed conduction, and increased electrogram fractionation. These changes were adjacent to EAT regions, suggesting a role for EAT in the pathogenesis of AF substrate (25). EAT often neighbored high dominant frequency (DF) sites, and pulmonary vein isolation plus EAT-based ablation was found to efficiently eliminate these high frequency sources and yield relatively high success in patients with persistent AF (26). EAT is thought to harbor ganglionated plexi (GP) which are believed to act as triggers for AF and promote AF maintenance through alterations in autonomic tone. In a study by Takahashi et al. (27), LA EAT overlapped 95% of the 5 major anatomic GP areas and corresponded to approximately 75% of CFAE sites.

Because conventional PVI transects three of four major atrial GPs as well as the ligament of Marshall, it is possible that PVI outcomes in arrhythmia suppression include the effect of autonomic denervation due to collateral ablation of the GP. Deliberate addition of GP ablation to conventional PVI yielded a higher success rate than either PVI or GP ablation alone in patients with paroxysmal AF (28). It is important to mention that the relationship uncovered in our study is between EAT volume and AF recurrence. The interface of EAT with the underlying atrial tissue, including spatial colocalization with atrial fibrosis and other features identified by electroanatomic mapping, were not assessed in this study. Whether tailoring ablation strategies based on the amount and distribution of LA EAT would be helpful in combatting AF recurrence after ablation procedures remains to be elucidated.

Our study has some limitations. First, it is an observational study without specific targeting of areas of overlap with epicardial fat. The optimal cutoff value we identified needs to be confirmed in a larger-scale multicenter study. Such validation would be crucial to delineate the role of EAT in AF recurrence before widespread clinical implementation as an approach for stratification of recurrence risk. EAT quantification was performed manually which is a time-consuming process prone to human error. AF recurrence was defined solely based on follow-up ambulatory monitoring ECGs, therefore any asymptomatic paroxysmal AF that may have occurred was not captured. Previously described predictors of AF recurrence like sleep apnea, heart failure and diabetes were not found to be associated with recurrence in our study, likely due to a lack of statistical power.

## Conclusion

In this study, we found that LA EAT, a marker with clear ties to AF pathophysiology, is an independent predictor of AF recurrence after ablation. Further studies are warranted to assess the exact mechanism by which EAT modulates the response to catheter ablation in AF.

## Data availability statement

The raw data supporting the conclusions of this article will be made available by the authors, without undue reservation.

## Author contributions

YC, KO, PB, and NA: conceptualization and methodology. YC, FM, and JK: data curation, formal analysis, and validation. YC, KO, and NA: investigation. YC, PB, and NA: writing—original draft. FM and JK: visualization. FM, JK, KO, PB, and NA: writing—review and editing. PB and NA: supervision. PB: project administration. NA: resources and funding acquisition. All authors have read and approved the final manuscript.

## Funding

This work was supported by the John Locke Charitable Trust to NA.

## Conflict of interest

The authors declare that the research was conducted in the absence of any commercial or financial relationships that could be construed as a potential conflict of interest.

## Publisher's note

All claims expressed in this article are solely those of the authors and do not necessarily represent those of their affiliated organizations, or those of the publisher, the editors and the reviewers. Any product that may be evaluated in this article, or claim that may be made by its manufacturer, is not guaranteed or endorsed by the publisher.

## Abbreviations

AF: atrial fibrillation
BMI: body mass index
BSA: body surface area
CA: catheter ablation
CT: computed tomography
EAT: epicardial adipose tissue
ECG: electrocardiogram
GP: ganglionated plexi
LA: left atrial
LGE: late gadolinium enhancement
MRI: magnetic resonance imaging
RF: radiofrequency
ROC: receiver operating characteristic.
